# *Streptacidiphilus durhamensis* sp. nov., isolated from a spruce forest soil

**DOI:** 10.1007/s10482-013-9938-9

**Published:** 2013-05-30

**Authors:** Patrycja Golinska, Lina Ahmed, Dylan Wang, Michael Goodfellow

**Affiliations:** 1 School of Biology, University of Newcastle, Newcastle upon Tyne, NE1 7RU UK; 2 Institute of Microbiology, Chinese Academy of Sciences, Beijing, 100101 People’s Republic of China; 3 Department of Microbiology, Nicolaus Copernicus University, 87 100 Torun, Poland

**Keywords:** Actinobacteria, Polyphasic taxonomy, *Streptacidiphilus durhamensis*, Spruce forest soil

## Abstract

The taxonomic position of three acidophilic actinobacteria, strains FGG38, FGG39 and FSCA67^T^, isolated from the fermentation litter layer of a spruce forest soil was established using a polyphasic approach. The strains were shown to have chemotaxonomic and morphological properties consistent with their classification in the genus *Streptacidiphilus* and formed a distinct phyletic line in the *Streptacidiphilus* 16S rRNA gene tree being most closely related to *Streptacidiphilus albus* DSM 41753^T^ (99.4 % similarity). DNA:DNA relatedness data showed that isolate FSCA67^T^ and the type strain of *S. albus* belonged to markedly distinct genomic species. The isolates had many phenotypic properties in common and were distinguished readily from their closest phylogenetic neighbours in the *Streptacidiphilus* gene tree using a broad range of these features. Based on the combined genotypic and phenotypic data the three isolates are considered to represent a new *Streptacidiphilus* species. The name *Streptacidiphilus durhamensis* sp. nov. is proposed for this taxon with isolate FSCA67^T^ (=DSM 45796^T^ = KACC 17154^T^ = NCIMB 14829^T^) as the type strain.

## Introduction

Acidophilic sporoactinobacteria which share key chemotaxonomic and morphological properties with streptomycetes grow between pH 3.5 and 6.5 with optimal growth at pH 4.5–5.5. These organisms are numerous and widely distributed in acidic habitats, notably coniferous soils (Williams et al. 1971; Khan and Williams 1975; Goodfellow and Dawson 1978; Cho et al. 2008) where they have a role in the turnover of fungal biomass (Williams and Robinson 1981). They are a source of antifungal compounds (Williams and Khan 1974) and produce chitinases and diastases with pH optima below those of neutrophilic streptomycetes (Williams and Flowers 1978). In a comprehensive numerical taxonomic study, Lonsdale (1985) found that acidophilic streptomycetes fell into several distinct clusters (taxospecies), which were clearly distinguished using a combination of phenotypic tests. Subsequent studies underpinned the taxonomic heterogeneity of the group (Goodfellow and Simpson 1987; Seong et al. 1993).

Representatives of three of the taxospecies circumscribed by Lonsdale (1985) were assigned to the novel genus *Streptacidiphilus* as *Streptacidiphilus albus*, the type species, *Streptacidiphilus carbonis* and *Streptacidiphilus neutrinimicus* by Kim et al. (2003). In the meantime, five additional species have been recognised: *Streptacidiphilus jiangxiensis* (Huang et al. 2004), *Streptacidiphilus oryzae* (Wang et al. 2006), *Streptacidiphilus anmyonensis*, *Streptacidiphilus melanogenes* and *Streptacidiphilus rugosus* (Cho et al. 2008) bringing the total validly named species to eight.

All but one of the *Streptacidiphilus* species formed a monophyletic lineage (clade 53) in the extensive phylogenetic study of the family *Streptomycetaceae* undertaken by Labeda et al. (2012), whilst the exception, *S. oryzae,* was found in the related clade 54. The taxonomic integrity of clade 53 was well supported by all three of the tree-making algorithms used by Labeda and his colleagues and was closely related to the genus *Kitasatospora*, the species of which formed a large statistically unsupported clade. The species of both of these genera fell within the evolutionary radiation occupied by the genus *Streptomyces* and hence in the final analysis may only be considered taxonomically valid if the genus *Streptomyces* is found to be truly paraphyletic. However, to date, it is notable that all streptacidiphili form either white or gray aerial hyphae which differentiate into flexuous to straight chains of spores with smooth surfaces on oatmeal agar (International *Streptomyces* Project [ISP] medium 3; Shirling and Gottlieb 1966).

In a continuation of our bioprospecting studies on acidophilic and aciditolerant filamentous actinobacteria isolated from a spruce forest soil, several representative isolates were found to have colonial properties typical of streptacidiphili. The aim of the present study was to establish the taxonomic status of three of these isolates using a polyphasic approach. The resultant data showed that the strains belong to a new *Streptacidiphilus* species, *Streptacidiphilus durhamensis* sp. nov.

## Materials and methods

### Organisms, maintenance and biomass preparation

Acidophilic and aciditolerant filamentous sporoactinobacteria were sought from the L, F, H, A1 and C horizons of the spruce forest soil at Hamsterley Forest, County Durham; details of the site and the dilution plate procedure used are given elsewhere (Golinska et al. 2013). Aliquots (100 μl) of serial dilutions were spread over the surfaces of oven dried plates of starch-casein medium (Kűster and Williams 1964) with agar (SCA) and gellan gum (GG) as gelling agents, the media were supplemented with cycloheximide and nystatin (each at 50 μg ml^−1^) and adjusted to pH 4.5 with 1 N HCl. The inoculated plates were incubated at 28 °C for 4 weeks, at which point *Streptacidiphilus*-like colonies were detected from suspensions prepared from each of the environmental samples. The 56 *Streptacidiphilus*-like isolates were subcultured from starch-casein media prepared using each of the gelling agents and found to grow well on starch-casein agar at pH 5.5 but poorly on this medium at pH 4.5 and 6.0 with no growth at pH 7.0. All of the isolates were maintained on acidified modified Bennett’s agar slopes (Jones 1949) at room temperature and as suspensions of mycelial fragments and spores in glycerol (20 %, w/v) at −80 °C. Three strains from the F-horizon, isolates FGG38, FGG39 and FSCA67^T^, were chosen for detailed taxonomic analyses.

Biomass for the molecular systematic study and most of the chemotaxonomic studies was prepared by cultivating the three isolates in shake flasks of yeast extract-malt extract broth (pH 5.5) at 150 revolutions per minute at 28 °C for 3 weeks. Cells were harvested by centrifugation and washed twice in distilled water; biomass for the chemotaxonomic analyses was freeze dried and that for the molecular systematic work stored at −20 °C. Biomass for the fatty acid analysis conducted on isolate FSCA67^T^ was harvested from modified Bennett’s broth (Jones 1949), adjusted to pH 5.5, after incubation at 28 °C for 7 days.

### Phylogenetic analyses

Extraction of genomic DNA, PCR-mediated amplification of the 16S rRNA genes of the three isolates and direct sequencing of the purified PCR products were carried out as described by Golinska et al. (2013). The search for the closest phylogenetic neighbours based on 16S rRNA gene similarly was performed using the EzTaxon server (http://eztaxon-e.ezbiocloud.net/, Kim et al. 2012). Phylogenetic analyses were carried out using MEGA 5 (Tamura et al. 2011) and PHYML (Guindon and Gascuel 2003) software packages following multiple alignment using Clustal W. Evolutionary distances were calculated and clustering determined using the maximum-likelihood, maximum-parsimony and neighbour-joining methods to generate phylogenetic trees. The topologies of the evolutionary trees were evaluated by a bootstrap analysis (Felsenstein 1985) derived from 1,000 resamplings of the neighbour-joining dataset using MEGA 5 software. The tree was rooted using the 16S rRNA gene sequence of *Streptomyces albus* subsp. *albus* DSM 40313^T^ (GenBank accession number AJ621602).

### DNA:DNA relatedness

DNA:DNA relatedness values (∆Tm) between isolate FSCA67^T^ and *S. albus* DSM 41753^T^ were determined in duplicate using a fluorimetric method (Gonzalez and Saiz-Jimenez 2005), the optimal temperature for renaturation (Tm) was calculated using the equation *Tor* − 0.51 (% GC) + 47. The melting temperature (Tm) at which 50 % of the initial double-stranded DNA denatured into single-stranded DNA for isolate FSCA67^T^ and the isolate FSCA67^T^: *S. albus* DSM 41753^T^ hybrid DNA preparations were compared and the differences (∆Tm) calculated.

### Chemotaxonomy

Isolates FGG38, FGG39 and FCSA67^T^ were examined for chemical markers known to be of value in the systematics of genera classified in the family *Streptomycetaceae* (Kämpfer 2012a). Standard chromatographic methods were used to determine the isomers of diaminopimelic acid (Staneck and Roberts 1974), isoprenoid quinones (Collins 1985), polar lipids (Minnikin et al. 1984) and whole-organism sugars (Hasegawa et al. 1983), using appropriate controls. Cellular fatty acids of isolate FSCA67^T^ were extracted, methylated and determined by gas chromatography (Hewlett Packard instrument 6890) and analysed using the standard Sherlock Microbial Identification (MIDI) system, version 5 (Sasser 1990). The G+C vol% of the DNA of this isolate was determined following the procedure described by Gonzalez and Saiz-Jimenez (2002).

### Cultural and morphological properties

The isolates were examined for cultural and morphological features following growth on standard media at 28 °C for 3 weeks. Cultural properties were determined using acidified tryptone-yeast extract, yeast extract-malt extract, oatmeal, inorganic salts-starch, glucose–asparagine, peptone-yeast extract-iron and tyrosine agars (ISP media 1–7, respectively; Shirling and Gottlieb 1966). The arrangements of hyphae and spore chains were detected on acidified oatmeal agar (ISP medium 3) after 14 days at 28 °C, using the coverslip technique of Kawato and Shinobu (1959). Spore arrangement and spore surface ornamentation of isolate FSCA67^T^ were observed by examining a gold-coated dehydrated preparation from the oatmeal agar plate with a scanning electron microscope (Cambridge Stereoscan 240) and the procedure described by O’Donnell et al. (1993).

### Phenotypic tests

An extensive range of phenotypic tests were carried out on all of the isolates using media and methods described by Williams et al. (1983), albeit with acidified media. The ability of the isolates to grow at various temperatures (10, 30, 35 and 40 °C), pH values (4, 5, 6 and 7) and NaCl concentrations (1, 3, 5, 7 and 10 %, w/v) was determined using acidified modified Bennett’s agar (Jones 1949).

## Results

### 16S rRNA gene sequencing and DNA:DNA relatedness studies

**Fig. 1:**
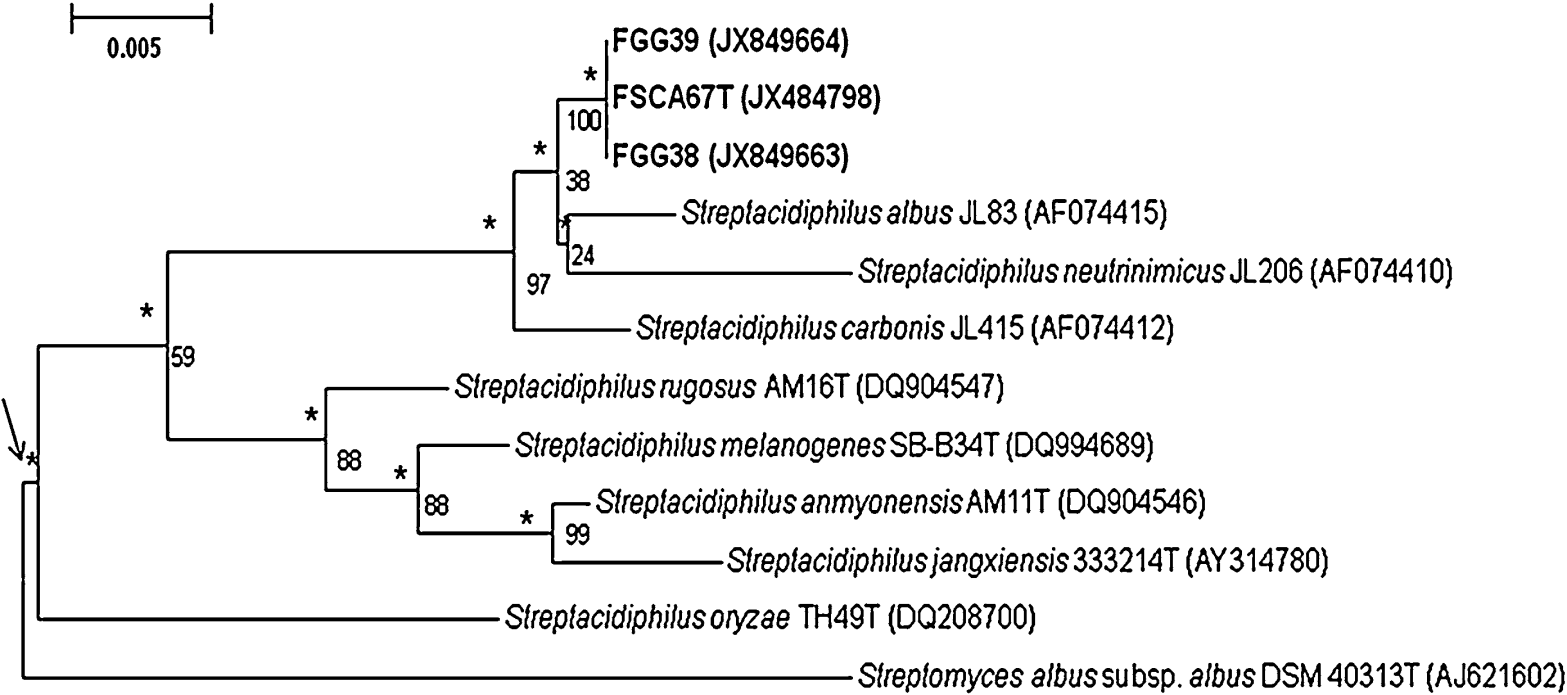
Neighbour-joining tree based on nearly complete 16S rRNA gene sequences (1,376–1,511 nucleotides) showing relationships between the isolates and the type strains of *Streptacidiphilus* species. *Asterisks* indicate branches that were also found using the maximum-likelihood and maximum-parsimony tree-making algorithms. *Numbers at the nodes* are percentage bootstrap values based on 1,000 re-sampled datasets, only values above 50 % are given. *T* type strain. *Bar* 0.005 substitutions per nucleotide position. The root position of the tree was determined using *Streptomyces* *albus* subsp. *albus* DSM 40313^T^ as outgroup

Near complete 16S rRNA gene sequences of isolates (1,423–1,463 nucleotides [nt]) FSCA67^T^, FGG38 and FGG39 were determined (GenBank accession numbers: JX484798, JX849663 and JX849664, respectively). The strains were found to have identical sequences and to form a branch in the *Streptacidiphilus* 16S rRNA gene tree that was shown to be supported by all of the tree-making algorithms and by a 100 % bootstrap value (Fig. 1). They were found to form a subclade in the *Streptacidiphilus* 16S rRNA tree together with the type strains of *S. albus*, *S. carbonis* and *S. neutrinimicus*, a taxon that was shown to be supported by a 97 % bootstrap value and all of the tree-making algorithms. The isolates were most closely related to *S. albus* JL83^T^ and found to share a 16S rRNA similarity with the latter of 99.4 %, a value found to correspond to 8 nt differences. The corresponding 16S rRNA gene similarities with the type strains of *S. carbonis* and *S. neutrinimicus* were shown to be 99.3 and 98.8 %, respectively, values equivalent to 10 and 17 nt differences. The 16S rRNA sequence similarities with the type strains of the remaining *Streptacidiphilus* species was found to be within the range 96.2–97.3 %. Notably, the 16S rRNA tree also supported the previously observed separation of *S. oryzae* from the other *Streptacidiphilus* species (Labeda et al. 2012).

The ∆Tm between isolate FSCA67^T^ g DNA and isolate FSCA67^T^: *S. albus* DSM 41753^T^ hybrid DNA was determined to be 9.5 °C (±2.83), a result that corresponds to a DNA similarity value of 45 % (Gonzalez and Saiz-Jimenez 2005), a value well below the 70 % cut-off point recommended for assigning strains to the same genomic species (Wayne et al. 1987).

### Chemotaxonomic, cultural, morphological and phenotypic properties

**Fig. 2:**
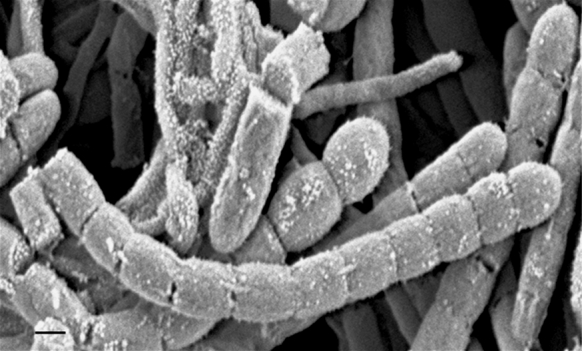
Scanning electron micrograph of isolate FSCA67^T^ showing straight chains of smooth-surfaced, cylindrical spores on oatmeal agar after growth for 3 weeks at 28 °C. *Bar* 0.5 μm

The isolates were found to produce an extensively branched substrate mycelium which carried abundant gray aerial hyphae that were shown to differentiate into straight to flexuous spore chains on oatmeal agar. Isolate FSCA67^T^ was found to produce long chains of smooth spores (Fig. 2). The isolates were shown to grow well on ISP media 2, 3 and 5; poorly on ISP 1; and not at all on ISP 4 (Table 1). In general, they were shown to form a variety of substrate mycelial pigments and, when formed, a white to medium gray aerial spore mass.

**Table 1:** Growth and cultural characteristics of isolates on ISP media after incubation for 3 weeks at 28 °C

Isolates	FGG38			FGG39			FSCA67^T^		
Medium	Growth	Colour of aerial spore mass	Colour of substrate mycelium	Growth	Colour of aerial spore mass	Colour of substrate mycelium	Growth	Colour of aerial spore mass	Colour of substrate mycelium
Yeast extract-malt extract agar (ISP 2)	++	Light gray	Yellowish brown	+++	Light gray	Yellowish brown	++	Light gray	Yellowish brown
Oatmeal agar (ISP 3)	+++	Medium gray	Yellowish gray	++	Medium gray	Light yellowish brown	+++	Medium gray	Light grayish yellowish brown
Glucose-asparagine agar (ISP 5)	++	Light gray	Moderate yellowish brown	+++	Light gray	Moderate yellowish brown	++	Light gray	Moderate yellowish brown
Peptone-yeast extract-iron agar (ISP 6)	+	−	−	−	−	−	−	−	−
Tyrosine agar (ISP 7)	+	Light gray	Moderate yellowish brown	+++	Light gray	Moderate yellowish brown	+	Light gray	Moderate yellowish brown

The isolates did not grow on medium 4 and grew poorly on ISP medium 1 forming a light yellow substrate mycelium and a white aerial spore mass

+++ abundant, ++ moderate, + poor, − no growth

**Fig. 3:**
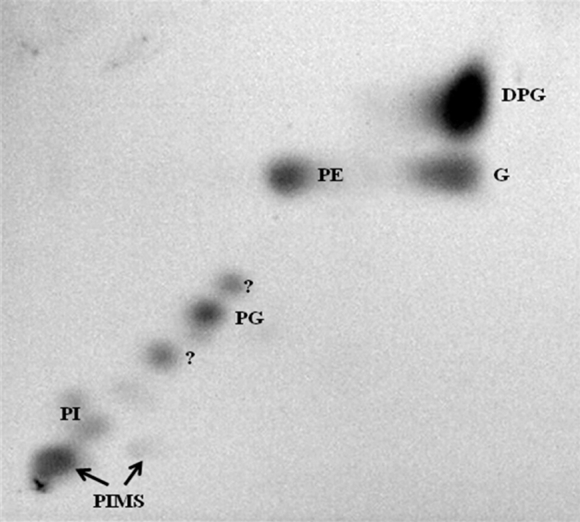
Two dimensional thin-layer chromatography of polar lipids of isolate FSCA67^T^ stained with molybdenum blue (Sigma). Chloroform:methanol:water (32.5:12.5:2.0 v/v) were used in the first direction and chloroform:acetic acid:methanol:water (40:7.5:6:2 v/v) in the second direction. *DPG* diphosphatidylglycerol, *PE* phosphatidylethanolamine, *PG* phosphatidylglycerol, *PI* phosphatidylinositol, *PIMS* phosphatidylinositol mannosides and *G* glycolipid

Whole-organism hydrolysates of the isolates were shown to be rich in ll-diaminopimelic acid (ll-A_2_pm) and to contain major amounts of galactose and rhamnose. The major polar lipids were established to be diphosphatidylglycerol, phosphatidylethanolamine, phosphatidylinositol and phosphatidylinositol mannosides, as exemplified in Fig. 3. The predominant isoprenologues of isolates FGG39, FGG38 and FSCA67^T^ were determined to be hexa- and octahydrogenated menaquinones with nine isoprene units, components found in ratios of 1:1.3, 1:1.4 and 1.8:1, respectively. The cellular fatty acid profile of isolate FSCA67^T^ was shown to consist of major proportions of (>10 %) of iso-C_15:0_ (15.8 %), anteiso-C_15:0_ (12.1 %) and iso-C_16:0_ (30.3 %), lower proportions (>2.8 %) of iso-C_14:0_ (6.2 %), iso-C_16:1_ H (3.4 %), C_16:0_ (9.1 %), anteiso-C_17:1_ ω9c (3.6 %), iso-C_17:0_ (2.8 %), anteiso-C_17:0_ (4.8 %) and C_17:0_ cyclo (3.4 %), summed features C_16:1_ ω7c/C_16:1_ ω6c (3.6 %) and iso-C_17:1_ ω9c/10 methyl C_16:0_ (2.8 %) plus trace amounts of other components (<0.5 %). The genomic G+C content of isolate FSCA67^T^ was 71.0 mol%.

The phenotypic properties of the isolates were compared with those of the type strains of *S. albus*, *S. carbonis* and *S. neutrinimicus* which had previously been studied using the same media and methods. It can be seen from Table 2 that the isolates can be distinguished from the type strains of their closest phylogenetic neighbours using a range of phenotypic features. Thus, the isolates, unlike *S. albus* DSM 41753^T^, their closest phylogenetic relative, were shown to metabolize Tween 40 and to grow on d-melezitose and d-xylose as sole carbon sources and at 10 and 30 °C. In contrast, the *S. albus* strain, unlike the isolates, degrades xanthine, uses sodium pyruvate and sodium propionate as sole sources of carbon and grows at pH 4.0. The isolates shared many other phenotypic features, as cited in the species description.

**Table 2:** Phenotypic properties that distinguish the isolates from the type strains of their nearest taxonomic neighbours

Characteristic	Isolates FGG38, FGG39 and FSCA67^T^	*S. albus* DSM 41753^T^	*S. carbonis* DSM 41754^T^	*S. neutrinimicus* DSM 41755^T^
Growth on acidified oatmeal agar				
Aerial spore mass	Medium grey	White	White	White/greyish white
Substrate mycelium	V (see Table 1)	Cream	Cream	Cream
Degradation of				
Tween 40	+	−	−	−
Tween 60	+	+	+	−
Xanthine	−	+	+	−
Growth on sole carbon sources				
At 1 %, w/v				
d-glucosamine	+	+	−	+
*Meso*-inositol	−	−	+	−
d-melezitose	+	−	−	−
l-rhamnose	+	+	+	−
d-xylose	+	−	+	+
At 0.1 %, w/v				
Sodium pyruvate	−	+	+	+
Sodium succinate	−	+	+	+
Growth on l-isoleucine (0.1 %, w/v) as sole nitrogen source	+	+	+	−
Growth at				
10 °C	+	−	−	−
30 °C	+	−	+	−
pH 4.0	−	+	+	+
pH 6.0	+	+	+	−
G+C content of DNA (mol %)	71	70–72	70–72	70–72

Data for the type strains of *S. albus*, *S. carbonis* and *S. neutrinimicus* was taken from Kim et al. (2003) and Cho et al. (2008)

+ positive, − negative

## Discussion

The three acidophilic isolates from the F-horizon of the spruce forest soil at Hamsterley Forest were found to share a range of cultural, chemotaxonomic and morphological properties typical of members of the genus *Streptacidiphilus* (Kim et al. 2003; Huang et al. 2004; Wang et al. 2006; Cho et al. 2008). They were shown to form extensively branched substrate mycelia, white to medium gray aerial hyphae that were found to differentiate into straight to flexuous chains of spores on oatmeal agar; and to have whole-organism hydrolysates rich in ll-A_2_pm, galactose and rhamnose, MK-9 (H_6_, H_8_) as the predominant isoprenologues; and diphosphatidylglycerol, phophatidylethanolamine (diagnostic lipid), phosphatidylinositol and phosphatidylinositol mannosides as major polar lipids (phospholipid pattern 2 after Lechevalier et al. 1977). Isolate FSCA67^T^ was shown to form long chains of smooth spores, contains fatty acids rich in saturated, iso- and anteiso- components (fatty acid type 2c; Kroppenstedt 1985) and a DNA G+C ratio of 71 mol%. The isolates were found to have identical 16S rRNA gene sequences and to form a subclade in the *Streptacidiphilus* 16S rRNA gene tree together with the type strains of *S. albus* (their nearest taxonomic neighbour), *S. carbonis* and *S. neutrinimicus*. DNA:DNA relatedness data showed that isolate FSCA67^T^ and *S. albus* DSM 41753^T^ belonged to distinct genomic species using the 70 % cut-off point recommended by Wayne et al. (1987). The isolates had identical phenotypic properties, some of which clearly distinguished them from the type strains of their closest phylogenetic neighbours. In light of all of these genotypic and phenotypic data, it is proposed that the isolates represent a new *Streptacidiphilus* species for which the name *S. durhamensis* is proposed with isolate FSCA67^T^ as the type strain. The species description is given below.

Filamentous spore-forming acidophilic actinobacteria remain a neglected group despite being common in acidic habitats (Williams et al. 1971; Khan and Williams 1975; Goodfellow and Dawson 1978; Goodfellow and Simpson 1987) and a potential source of novel acid-stable antibiotics and enzymes (Williams and Khan 1974; Williams and Flowers 1978; Williams and Robinson 1981). The present study provides further evidence that the genus *Streptacidiphilus* is underspeciated and common in acidic habitats, notably in coniferous litter (Lonsdale 1985; Goodfellow and Simpson 1987; Golinska, *unpublished observations*). Indeed, like isolates FGG38, FGG39 and FSCA67^T^, two out of the eight validly named *Streptacidiphilus* species were isolated from a single location, the spruce forest soil at Hamsterley Forest, County Durham. Additional comparative taxonomic surveys of streptacidiphili isolated from diverse acidic habitats can be expected to throw additional light on the evolutionary radiation of these organisms and thereby help underpin the continued recognition of the genus or indicate whether it should be seen as a genus *incertae sedis* (Kämpfer 2012b).

### Description of *Streptacidiphilus durhamensis* sp. nov.

*Streptacidiphilus durhamensis*. N.L. masc. adj. *durhamensis*, belonging to Durham, a country in the North East of England, the source of the isolates.

Aerobic, Gram-positive, non-acid alcohol fast, acidophilic actinobacteria which form an extensively branched substrate mycelium that carries aerial hyphae which differentiate into long straight to flexuous chains of smooth, cylindrical spores (0.7 × 1.0 μm). Grows from 10 to 30 °C, optimally ~26 °C, from pH 4.5–6.0, optimally ~pH 5.5, and in the presence of 1 %, but not 3, 5, 7 and 10 %, w/v NaCl. Aesculin and arbutin are hydrolysed, but not allantoin or urea. Does not reduce nitrate. Degrades starch, but not adenine, casein, chitin, elastin, guanine, l-tyrosine or xylan. d-arabitol, l-arabinose, d-cellobiose, d-fructose, d-galactose, d-glucosamine, d-glucose, d-glycerol, d-lactose, d-mannitol, d-raffinose, d-salicin, d-sucrose and d-trehalose are used as sole carbon sources for energy and growth, but not l-arabitol, dextran, *meso*-erythritol, d-glucosamine or xylitol (all at 1 %, w/v). Does not use acetate, adipate, benzoate, butyrate, citrate, fumarate, hippurate, oxalate or propionate (sodium salts) or *para*-hydroxybenzoic acid as sole carbon sources (all at 0.1 %, w/v). l-alanine, l-*iso*leucine, l-phenylalanine and l-valine are used as sole nitrogen sources, but not l-arginine, l-cysteine or l-histidine (all at 0.1 %, w/v). Aminoethanol, l-cyteine, l-hydroxyproline, l-*iso*leucine, l-phenylalanine, l-serine and l-valine are used as sole carbon and nitrogen sources, but not acetamide, l-histidine or l-methionine (all at 0.1 %, w/v). Additional phenotypic properties are cited in Tables 1 and 2. The major fatty acids of the type strain are anteiso-C_15:0_, iso-C_15:0_ and iso-C_16:0_. The remaining chemotaxonomic markers are typical of the genus *Streptacidiphilus*. The G+C content of the DNA of the type strain is 71.0 mol%.

The species currently contains the type strain FSCA67^T^ (=DSM 45796^T^ = KACC 17155^T^ = NCIMB 14829^T^) and strains FGG38 and FGG39, all of which were isolated from the F-horizon of a *Picea sitchensis* forest at Hamsterley Forest, County Durham, England.
